# Analysis of Factors That Determine Weight Gain during Smoking Cessation Therapy

**DOI:** 10.1371/journal.pone.0072010

**Published:** 2013-08-21

**Authors:** Maki Komiyama, Hiromichi Wada, Shuichi Ura, Hajime Yamakage, Noriko Satoh-Asahara, Akira Shimatsu, Hiroshi Koyama, Koichi Kono, Yuko Takahashi, Koji Hasegawa

**Affiliations:** 1 Department of General Internal Medicine, National Hospital Organization Kyoto Medical Center, Kyoto, Japan; 2 Division of Translational Research, National Hospital Organization Kyoto Medical Center, Kyoto, Japan; 3 Clinical Research Institute, National Hospital Organization Kyoto Medical Center, Kyoto, Japan; 4 Department of Hygiene and Public Health, Osaka Medical College, Osaka, Japan; 5 Health Care Center, Nara Women’s University, Nara, Japan; University of Santiago de Compostela School of Medicine - CIMUS, Spain

## Abstract

Cigarette smokers are generally known to gain weight after quitting smoking, and such weight gain is thought to contribute to the worsening of glucose tolerance. While smoking cessation therapy such as nicotine replacement is useful to minimize post-cessation weight gain, substantial gain occurs even during the therapy. The purpose of the present study was to identify factors associated with weight gain during smoking cessation therapy. We evaluated 186 patients(132 males and 54 females)who visited our outpatient clinic for smoking cessation, and successfully achieved smoking abstinence. We performed gender-adjusted regression analysis for the rate of BMI increase from the beginning of cessation to 3 months after initiation. Furthermore, we performed multivariate analysis to investigate factors that determine the BMI increase after smoking cessation. The mean BMI significantly (p<0.0001) increased from 23.5±3.6 kg/m^2^ at the initial consultation to 23.9±3.8 kg/m^2^ at 3 months after the start of therapy. There was no significant difference in the extent of BMI increase between nicotine patch and varenicline therapy groups. Factors significantly correlated with the %BMI increase at 3 months after the start of therapy were triglyceride (p = 0.0006, **β**a = 0.260), high-density lipoprotein cholesterol (p = 0.0386, βa = −0.168), daily cigarette consumption (p = 0.0385, βa = 0.154), and the Fagerström Test for Nicotine Dependence (FTND) score (p = 0.0060, βa = 0.203). Stepwise multivariate analysis demonstrated that triglyceride and the FTND score were the factors determining the post-cessation BMI increase and that the FTND score was the strongest one. The present study demonstrated that smokers with a high FTND score are more likely to gain weight during smoking cessation therapy. Thus, smokers with a high nicotine dependency may require intervention against weight gain in the cessation clinic.

## Introduction

Cessation of smoking lowers the risk of fatal diseases such as cancer and cardiovascular disease, and reduces all-cause mortality [1]–[5]. Yet it is also known that one gains weight after quitting smoking [6]. Regarding the extent of such weight gain, various reports exist, but it is considered that, for persons who quit smoking on their own initiative, on average, males gain 2.8 kg, females gain 3.8 kg, and over 10% of persons gain 13 kg or more [7]. It has been reported that the body weight increases for around three years after ceasing to smoke, while, thereafter, it decreases, and, at seven or eight years after quitting, the body weight returns to the pre-cessation level [8]. Although mechanisms leading to post-smoking cessation weight gain are not well understood, the following are considered: an increase in caloric intake [9], [10], a decline in resting-state basal metabolism [9], decreases of physical activity [9], [11], [12], and increase in lipoprotein lipase activity [9], [13], [14]. A variety of other important factors have also been suggested. These include the elimination of appetite suppression by nicotine [15], improvements in taste and smell perception, a desire to have something in the mouth, and improvements of the gastric mucosal microcirculation.

It has been reported that, for around three years after ceasing to smoke, there is short-term worsening of glucose intolerance [16], [17]. People with a higher post-smoking cessation weight gain have a greater risk of glucose intolerance [16], [18]. Other damage from post-cessation weight gain includes a reduction in the beneficial effects on pulmonary function [19], and the fact that worries about the weight gain might lead to a failure to quit smoking [20], [21]. Amelioration of post-smoking cessation weight gain leads to the improvement of glucose intolerance [22]. The joint use of nicotine replacement therapy and diet and exercise therapy not only helps to prevent post-cessation weight gain [23], [24], but is also linked to higher smoking cessation rates [25]. However, substantial body weight gain still occurs during smoking cessation therapy in outpatient clinics, and such weight gain is associated with an increase in the level of classical atherosclerotic risk factors [26], [27].

Factors to help predict post-cessation body weight gain have been reported in the past. These factors include being a heavy smoker, of African-American descent, and young [28]. However, within smoking-cessation outpatient clinics, factors that predict post-cessation weight gain at the initial evaluation prior to smoking-cessation therapy have not been elucidated. If one could pre-select patients who require intervention against weight gain during the initial stage of outpatient smoking-cessation sessions, it may be possible to prevent a worsening of glucose intolerance, as well as to boost smoking-cessation success rates. To investigate how to predict prior to therapy those likely to show an increase in body weight after smoking-cessation therapy, the present study investigated factors that are involved in post-smoking cessation body-weight gain.

## Methods

### Subjects

We analyzed 186 consecutive patients (132 males and 54 females, aged between 22 and 81 years) who visited the Smoking Cessation Clinic, National Hospital Organization Kyoto Medical Center between July 2007 and November 2011, and successfully quit smoking. Among the 186 patients, 50 received antihypertensive agents, 27 received statins, and 21 received agents against diabetes mellitus. Various parameters were evaluated in these patients at the time of the initial consultation and after quitting smoking (at 12 weeks after the initial consultation).

This study was performed after explaining the objective to the subjects in writing and obtaining their consent. All participants provided their written informed consent. The protocol was approved by the Ethical Review Board, National Hospital Organization Kyoto Medical Center.

### Evaluation of Depression

The severity of depression was evaluated using a questionnaire based on the self-rating depression scale (SDS). The questionnaire was answered by the patients themselves, the answers were checked by the study staff, and the patients were asked to fill it in again if there were items that the subjects omitted or mistakes had been made on answering [29], [30].

### Blood Sampling

Blood was obtained from each patient’s antecubital vein 2–3 hours after lunch to determine hemoglobin A1c (HbA1c), triglyceride (TG), low-density lipoprotein-cholesterol (LDL-C), high-density lipoprotein-cholesterol (HDL-C), and high sensitivity C-reactive protein (hsCRP) levels.

### Smoking Cessation Clinic

At the initial consultation, nicotine dependence was assessed with the Fagerström Test for Nicotine Dependence (FTND) [31]. In this test, scores range from 0 to 10, with higher scores indicating more severe nicotine dependence. The number of cigarettes smoked per day was determined by asking the smoker, “On average, in the past month, how many cigarettes did you smoke per day?” Smokers were asked to rate their confidence in their ability to abstain from smoking cigarettes over the next 3 months on a scale from 0 to 100%.

Anti-smoking treatment was conducted according to the Standard Procedures for Anti-Smoking Treatment (originally issued in March 2006 by the Japanese Circulation Society, Japan Lung Cancer Society, and Japanese Cancer Association). The patients were examined on their first visit and 2, 4, 8, and 12 weeks thereafter and treated with nicotine patches or the oral administration of varenicline. On their repeated visits, whether or not the patients had maintained smoking cessation was checked, and specific advice concerning the continuation of cessation was given. At the end of the anti-smoking treatment (after 12 weeks), whether or not cessation had been maintained was evaluated. A patient was judged to have succeeded in quitting smoking with an expiratory carbon monoxide (CO) concentration of 7 ppm or less and the patient’s affirmation of no smoking. The attempt to quit smoking was judged to have been unsuccessful when the patient stopped visiting during the treatment period or continued visiting but failed to quit smoking.

### Statistical Analysis

Data are presented as the mean ± SD, and p<0.05 was considered significant. Statistical analyses were performed as previously described [32]. Data on various parameters of smoking patients before and after successful quitting smoking were compared by the Mann-Whitney U-test. The gender-adjusted correlations of various parameters were examined according to Pearson’s correlation coefficient. Factors that determine weight gain after smoking cessation were analyzed using multiple logistic regression analysis.

The power of multiple regression analysis was calculated using post-hoc statistical power analysis. At that time, the power was calculated using the sample size, number of independent values, and actually obtained R^2^. The type I error probability (α) was 0.05.

## Results

The average age of participants was 59.6±12.5 years, the daily number of cigarettes smoked was 23.5±11.2, the Brinkman index was 883±490, CO concentration in exhaled breath was 16.9±18.1 ppm, the SDS score was 37.3±10.2, and the FTND score was 6.8±1.9. The breakdown of smoking-cessation therapies for participants was as follows: varenicline, 95 patients; a nicotine patch, 89 patients; and no therapeutic medication, two patients. Table 1 shows data at the time of the first examination and after smoking cessation (at 12 weeks after the first examination). After the cessation of smoking, compared with the initial examination, the BMI increased (p<0.001), systolic blood pressure decreased (p = 0.011), TG increased (p = 0.002), LDL-C increased (p = 0.019), and HDL-C increased (p<0.001).

**Table 1:** Data on patients before and after successful smoking cessation.

	Before	After	*p-* value
BMI (kg/m^2^)	23.5±3.6	23.9±3.8	<0.001
SBP (mmHg)	130±20	127±20	0.011
DBP (mmHg)	75±11	74±12	0.104
HbA1c (%)	5.7±1.0	5.7±0.9	0.757
TG (mg/dl)	173±105	199±129	0.002
LDL-C (mg/dl)	116±28	120±30	0.019
HDL-C (mg/dl)	54.2±15.5	57.8±16.5	<0.001

Data are presented as the mean ± SD.

Next, we compared pre- and post-cessation changes between nicotine patch and varenicline treatment groups. Table 2 shows basic data for each separate group at the time of the first examination. Significant differences were recognized for age–the nicotine patch group was older (P<0.05)–and, for the number of cigarettes smoked per day and FTND, respectively, the varenicline group was higher. Table 3 shows the results of comparing pre- and post-cessation changes for each therapy group. The HDL-C level increased in both nicotine-patch and varenicline therapy groups. The SBP declined in the varenicline group, and TG and LDL-C increased in the nicotine patch group. Regarding the BMI increase, both groups showed the same level, 0.4 kg/m^2^, and, thus, no significant difference was recognized between them. Moreover, for categories other than the BMI, no significant inter-therapy group differences were recognized for pre- and post-smoking cessation changes.

**Table 2:** Data on patients before smoking cessation therapy.

	Nicotine Patch	Varenicline
	(n = 89)	(n = 95)
Male/female	67/22	63/32
Age (years)	61.6±13.4	57.6±11.5#
Daily cigarette consumption (n)	21.7±10.5	25.2±11.9#
Brinkman index	814±455	935±524
CO (ppm)	15.6±10.6	19.0±17.2
SDS test score	37.0±9.6	37.5±10.4
FTND score	6.7±1.9	7.5±1.8##

# *P*<0.05,

## *P*<0.01 vs. “Nicotine Patch”.

**Table 3:** Data on patients before and after smoking cessation therapy.

	Nicotine Pacth		Varenicline		*P-* value†
	(n = 89)		(n = 95)		time × group
	Before	After	Before	After	
BMI (kg/m^2^)	23.6±3.4	24.0±3.8**	23.4±3.8	23.8±3.9**	0.716
SBP (mmHg)	130±19	128±19	130±21	125±21*	0.358
DBP (mmHg)	75±11	73±12	76±12	74±13	0.777
HbA1c (%)	5.7±0.8	5.8±0.9	5.6±1.1	5.6±1.0	0.639
TG (mg/dl)	173±106	205±133**	171±102	194±126	0.591
HDL-C (mg/dl)	53.5±15.6	56.6±16.4**	55.2±15.5	59.1±16.8**	0.623
LDL-C (mg/dl)	114±28	120±30*	118±28	119±30	0.225

* *P*<0.05,

** *P*<0.01 vs. before smoking cessation therapy.

† Two-way repeated measures ANOVA (time [before and after] × group).

Table 4 shows the results of regression analysis adjusted for gender of BMI change rates. Concerning evaluation categories at the time of the initial examination, the TG (p = 0.0006, **β**a = 0.260), HDL-C (p = 0.0386, **β**a = −0.168), number of cigarettes smoked per day (p = 0.0385, **β**a = 0.154), and FTND score (p = 0.0060, **β**a = 0.203) were significantly correlated with the rates of the post-smoking cessation BMI increase. To further investigate factors involved in the post-cessation BMI increase, we performed multivariate analysis. The results demonstrated that the TG and FTND score were factors determining the post-cessation BMI increase, and that the FTND score was the strongest one.

**Table 4:** Gender-adjusted regression analysis for the change of BMI.

	Change of BMI (%)	
	*β* a	*p*
Univariate		
Age	−0.075	0.3284
BMI (kg/m2)	0.068	0.3600
SBP (mmHg)	0.145	0.0527
DBP (mmHg)	0.143	0.0576
HbA1c (%)	−0.045	0.5569
TG (mg/dl)	0.260	0.0006
HDL-C (mg/dl)	−0.168	0.0386
LDL-C (mg/dl)	0.084	0.2723
hsCRP (log)	−0.184	0.3232
Daily cigarette consumption (n)	0.154	0.0385
Duration of smoking (years)	−0.082	0.3198
Brinkman index	0.121	0.1192
CO (ppm)	−0.003	0.9630
SDS test score	−0.027	0.7246
FTND score	0.203	0.0060
Stepwise multivariate analysis	(*r* ^2^ = 0.084)	
TG	0.187	0.0109
FTND score	0.236	0.0019

*β*: Standardized coefficient.

a Gender-adjusted.

Within the multiple regression analysis of Table 4, R^2^ was 0.084. At that time, there were three independent variables (gender, TG, and FTND), and the sample size was 186; therefore, the power was calculated to be 0.994, confirming a large value.

Investigation was conducted to assess whether a patient group susceptible to post-smoking cessation weight gain could be specified by FTND scores at the initial smoking-cessation examination. As shown in Table 5, there was a “stepping up” of BMI increases rates between FTND 7 and 8, based on which subjects were divided into two groups:one with FTND scores of 7 or less, and one with FTND scores of 8 or more, and comparisons were made of pre- and post-smoking cessation changes. The group with FTND scores of 7 or less consisted of 103 persons (73 males, 30 females, mean age: 62.9±12.0 years), daily number of cigarettes smoked: 18.9±7.5, Brinkman index: 737±364, CO concentration of exhaled breath: 13.5±8.9 ppm, SDS test score: 35.5±8.9, and FTND scores: 5.7±1.3. The group with FTND scores of 8 or more had 80 persons (56 males, 24 females, mean age: 55.1±12.0 years), daily number of cigarettes smoked: 30.3±13.0, Brinkman index: 1082±579, CO concentration of exhaled breath: 22.3±18.4 ppm, SDS test score: 39.2±10.7, FTND scores 8.9±0.7.

**Table 5:** Changes in BMI for respective FTND scores (mean ± standard deviation).

	Change of BMI (%)
	Mean±SD
FTND	
<5	0.3±3.2
5	0.8±3.0
6	1.5±4.9
7	1.1±4.0
8	2.0±3.2
9	2.4±3.6
10	3.4±4.0

As shown in Table 6, although there was a significant decline in the pre- to post-cessation diastolic blood pressure within the FTND 7 or less group, no changes were seen in the FTND 8 or more group. Regarding serum HDL-C values, while a significant increase was observed in the FTND 8 or more group, no changes were seen in pre- to post-cessation HDL-C in the FTND 7 or less group. Concerning the body weight, although a significant BMI increase was noted in the FTND 7 or less group, the changes were minimal. Conversely, in the FTND 8 or more group, there was a larger post-cessation BMI increase, and a significant difference in the level of BMI increase was noted in this group compared with the FTND 7 or less group. Regarding the level of changes from pre- to post-cessation, other than BMI, no other items showed a significant difference between the FTND 7 or less and FTND 8 or more groups.

**Table 6:** Pre- and post-smoking cessation data based on FTND scores.

	FTND ≦ 7.0		8.0 ≦ FTND		
	(n = 103)		(n = 80)		*P-*value†
	Before	After	Before	After	Time × Group
BMI (kg/m^2^)	23.3±3.4	23.5±3.6**	23.8±3.8	24.4±4.1**	0.006
SBP (mmHg)	129±19	126±20	132±20	128±20	0.894
DBP (mmHg)	75±11	72±12**	76±11	76±12 #	0.018
HbA1c (%)	5.7±1.0	5.7±0.8	5.6±0.8	5.7±1.1	0.131
TG (mg/dl)	160±98	186±115*	192±113	220±145*	0.883
HDL-C (mmol/l)	55.2±15.2	57.9±16.1**	52.7±15.6	57.3±16.8**	0.225
LDL-C (mmol/l)	114±26	119±29*	119±29	122±30	0.631

Data are presented as the mean ± SD.

* *P*<0.05, **P<0.01 vs. before measurement.

# *P*<0.05 vs. “FTND ≦ 7.0”.

† Two-way repeated measures ANOVA (time [before and after] × group).

## Discussion

In the present study, although a significant increase in BMI was confirmed after smoking-cessation therapy, the BMI increase was only 0.4 kg/m^2^ (1.1 kg), which is much smaller than reported in previous studies for people who quit smoking on their own initiative (2.8–3.8 kg) [7]. The following reasons can be considered. Firstly, observation was only a short period of three months in an outpatient clinic for smoking cessation. Secondly, previous studies reported that a young age is a factor in post-cessation weight gain [28]; however, patients in the present study were outpatients with a relatively older average age of 60 years. Thirdly, it is possible that the use of medications such as nicotine patches and varenicline contributed to the suppression of weight gain. In a previous study, it was reported that, where varenicline was used in smoking cessation, weight gain after one year was 0.4 kg, while, with a nicotine patch, it was 0.5 kg [33]–thus, weight gain during smoking cessation therapy appears to be low compared with ceasing smoking on one’s own initiative [7]. In the nicotine-replacement therapy, a nicotine-induced decrease in appetite and increase in basal metabolism are thought to be the mechanism for weight-gain suppression. In detail, nicotine not only acts on the lateral hypothalamic area to suppress appetite [34], but also works to suppress neuropeptide Y, a peptide which increases food intake that is located in the arcuate nucleus of the hypothalamus [35]. Further, the activation of nicotine receptors stimulates the expression of uncoupling protein 1 within white and brown adipocytes, which results in a rise in basal metabolism [36]. Recently, it was reported that the forced activation of hypothalamic AMP-activated protein kinase inhibits these effects of nicotine. In other words, the effects of nicotine, such as appetite suppression and induction of uncoupling protein 1 expression in adipose tissue, are mediated by the inactivation of hypothalamic AMP-activated protein kinase [37]. As for varenicine, after it couples within nicotine receptors in the brain, dopamine is released, which is suggested to be connected with the suppression of appetite.

In the present study, no significant differences in body-weight increases were recognized for the different therapeutic methods. However, in the basic data for pre-smoking cessation, the varenicline group had a higher nicotine dependency than the nicotine-patch group. Considering that a higher nicotine dependency is associated with a larger increase in the BMI, we cannot rule out the possibility that varenicline led to a relatively stronger suppression of body-weight gain than the nicotine patch. Further studies are needed regarding weight-gain for each different therapeutic method.

In a previous study, the characteristics of smokers who showed post-smoking cessation weight gain included persons who smoked large numbers of cigarettes per day, African-Americans, and young persons [28].

From the results of multivariate analysis, it was determined that, from among the items analyzed at the time of the initial examination, FTND was the most important factor regulating post-smoking cessation body-weight gain. The result that a high FTND score (i.e., a strong nicotine dependency) was the most important determinant of a BMI increase supports the hypothesis that post-cessation weight gain is one of the nicotine withdrawal symptoms. Serum TG elevation was also related to post-cessation body-weight gain, but the reasons for this are not clearly understood. Although TG levels are generally correlated with BMI, from the results of multivariate analysis, BMI at the first consultation was not a determinant of the post-cessation BMI increase (β^a^ = −0.045, p = 0.562). Nicotine does suppress lipolysis via adipose tissue lipoprotein lipase (LPL) inhibition. At the same time, due to the suppression of neutral fat uptake, net energy expenditure within adipose tissue is adjusted to become negative [38]. The more cigarettes a patient smokes, the higher the TG serum levels become [39]. In such patients, LPL activity that had been suppressed undergoes a marked increase after smoking-cessation, resulting in a tendency towards positive energy expenditure within adipose tissue, which may lead to body-weight gain. However, further studies are required of the precise mechanisms by which serum TG elevation in smokers leads to post-smoking cessation weight gain. It is also thought that serum TG elevation within the initial examination period could possibly have involved dietary practices, including the intake of high-carbohydrate foods, and excessive alcohol consumption. Smokers with dietary practices that can easily lead to visceral fat obesity may experience larger body-weight gains due to an increased appetite after smoking cessation.

In the present study, in addition to the regression analysis results for FTND and TG, the number of cigarettes smoked per day, and lowness of HDL-C were correlated with post-smoking cessation BMI elevation. It is considered that the number of cigarettes smoked per day is negatively correlated with serum HDL-C [40]. Further, the number of cigarettes smoked is consistent with previous reports, and thought to contribute to post-cessation weight gain mediated by the level of nicotine dependence. In addition to this, however, the desire to have something in the mouth may also be involved.

It also cannot be denied that other strong determining factors may exist in items not tested within the present study.

In the results of multivariate analysis, the R-square value was low. The reasons are thought to be:firstly, that weight gain itself in the present study was low;Secondly, in addition to the elimination of nicotine-induced appetite suppression, a variety of factors are thought to have been involved that could not be predicted only from the items analyzed in this study at the time of the initial examination. Namely, improvements in taste and smell perception, improvements of circulatory blockages in the gastric mucosal microcirculatory system, an increase of food intake due to the desire to have something in the mouth, a decline in resting-state basal metabolism, and decreases in the level of physical activity. As daily-life habits such as eating and exercise patterns strongly influence insulin resistance, further large-scale studies will be necessary regarding the relationships between post-smoking cessation weight gain and daily-life habits.

Recently, the relationship between post-smoking cessation weight gain and cardiovascular events was reported. When a person without diabetes has stopped smoking for more than four years, even if there is weight gain, the cardiovascular risk is still substantially reduced compared with current smokers, and the longer the non-smoking period, the lower the cardiovascular disease incidence rate. However, it is suggested that, for a person with diabetes, in order to clearly demonstrate smoking-cessation effects, that individual must keep post-smoking cessation body-weight gain to under 5 kg as well as keep smoking abstinence for over four years [41].

Even if one is expected to experience post-cessation weight gain, quitting smoking still leads to a reduced cardiovascular risk. However, there is also a possibility that if one can prevent post-cessation weight gain, then this will further reduce the cardiovascular risk due to having ceased smoking. Body-weight gain itself is considered a factor that hinders the desire to quit smoking. From these considerations, for effective smoking-cessation treatment, at the initial outpatient examination for smoking cessation, one must determine the patients expected to gain weight after ceasing smoking, and perform weight control accordingly. This may possibly help to prevent post-cessation weight gain, and the results of the present study may be useful for discriminating such patient groups.

It has been reported that lipoprotein lipase–which works to incorporate neutral fat into cells–increases for around six months after smoking ceases [14], [42]. Compatible with this, an increase in appetite occurs predominantly in the first six months after ceasing smoking and continues until around one year after cessation. Diet and exercise therapy are thought to prevent post-smoking cessation weight gain [24]. Considering that an appetite increase is one of the factors in nicotine withdrawal syndrome, a strict diet during smoking cessation therapy might interfere with ceasing smoking. Further studies are needed regarding at what timing we should perform intervention against post-smoking cessation weight gain.
